# Intimate partner violence against women in Nigeria: a multilevel study investigating the effect of women’s status and community norms

**DOI:** 10.1186/s12905-018-0628-7

**Published:** 2018-08-09

**Authors:** Faith Owunari Benebo, Barbara Schumann, Masoud Vaezghasemi

**Affiliations:** Epidemiology and Global Health Unit, Department of Public Health and Clinical Medicine, Umea University, SE -901 87 Umea, Sweden

**Keywords:** Intimate partner violence, women’s status, Community norms, Multilevel analysis, Nigeria

## Abstract

**Background:**

Intimate partner violence (IPV) against women has been recognised as a public health problem with far-reaching consequences for the physical, reproductive, and mental health of women. The ecological framework portrays intimate partner violence as a multifaceted phenomenon, demonstrating the interplay of factors at different levels: individual, community, and the larger society. The present study examined the effect of individual- and community-level factors on IPV in Nigeria, with a focus on women’s status and community-level norms among men.

**Methods:**

A cross-sectional study based on the latest Nigerian Demographic Health Survey (2013) was conducted involving 20,802 ever-partnered women aged 15–49 years. Several multilevel logistic regression models were calibrated to assess the association of individual- and community-level factors with IPV. Both measures of association (fixed effect) and measures of variations (random effect) were reported.

**Results:**

Almost one in four women in Nigeria reported having ever experienced intimate partner violence. Having adjusted for other relevant covariates, higher women's status reduced the odds of IPV (OR = 0.47; 95% CI = 0.32–0.71). However, community norms among men that justified IPV against women modified the observed protective effect of higher women's status against IPV and reversed the odds (OR = 1.89; 95% CI = 1.26–2.83).

**Conclusions:**

Besides women’s status, community norms towards IPV are an important factor for the occurrence of IPV. Thus, addressing intimate partner violence against women calls for community-wide approaches aimed at changing norms among men alongside improving women’s status.

**Electronic supplementary material:**

The online version of this article (10.1186/s12905-018-0628-7) contains supplementary material, which is available to authorized users.

## Background

Intimate partner violence (IPV) is a global concern with a significant public health impact [1, 2]. The World Health Organization (WHO) defines IPV as any behavior within an intimate relationship by an intimate partner that causes physical, psychological, or sexual harm to those in the relationship. It is one of the most common types of violence experienced by women [1, 3]. Most reported cases of IPV are perpetrated by men towards women [1]; although men can be victims of IPV, this paper focuses on women. Violence against women is associated with immediate and long-term adverse health outcomes for women and children, both directly and indirectly [2]. In a WHO multi-country study, women who had experienced IPV reported poorer health, more emotional distress, and more suicidal thoughts and attempts than those who had not experienced IPV [4]. Two in three victims of intimate partner/family-related homicide are women [2, 5]. IPV limits a woman’s decision-making power regarding her reproductive health, putting her at risk for sexually transmitted infections (STIs) and unwanted pregnancies. Partner violence during pregnancy can be associated with poor attendance to antenatal and postnatal care, increasing the risk of having low birth-weight infants or preterm births and intensive care admission of the newborn [1, 5].

Globally, over a third (35%) of women have experienced physical and/or sexual violence by an intimate partner or sexual violence by a non-partner at some point in their lives [2, 6]. A WHO report on global and regional estimates of violence against women found that the global lifetime prevalence of IPV among ever-partnered women was 30%, and for Africa 37% [2]. Reports from the Nigerian national population commission estimated women’s lifetime exposure to IPV from their current husband or partner at 19% for emotional IPV, 14% for physical IPV, and 5% for sexual IPV [6]. Previous studies from Nigeria have shown the prevalence of IPV to range from 31 to 61% for psychological/emotional violence, 20 to 31% for sexual violence, and 7 to 31% for physical violence [7]. Furthermore, studies conducted in different regions in Nigeria have reported prevalence of IPV ranging from 42% in the North [8], 29% in the South West [9], 78.8% South East [10], to 41% in the South South [11].

Researchers have proposed different theories and frameworks to explain and understand violence against women. These are important, as they can guide the design of effective prevention and intervention strategies. Scholars argue that violence against women is an expression of patriarchal domination of women by men, rooted in gender and power inequality [12]. In some societies, men are the breadwinners, while women are expected to be homemakers, to care for the children, and to be economically dependent on the men. When changes occur in the traditional gender order and roles, violence can result, particularly in patriarchal societies [13–15]. For example, if a wife disobeys or challenges her husband or does not play her gendered role, the husband may resort to violence to discipline her. He does this to put her in her place and to maintain his power and control [13, 15].

The ecological framework [16] portrays partner violence as a multifaceted phenomenon with embedded levels of causality and demonstrates the interplay of factors at different levels: individual, community, and the larger society. This framework illustrates that a single factor is neither sufficient nor necessary for partner violence to occur [13, 16]. Inspired by this framework, the present study examined the effect of individual- and community-level factors on IPV in Nigeria, with a focus on women’s status and community norms.

Women’s status is a complex phenomenon and it varies between societies and social locations such as household, neighborhood, community and the larger society. Factors that may enhance women’s status in one context may be detrimental in another [17, 18]. Some terms and concepts that have been used in the literature to assess women’s status include female autonomy**,** women empowerment**,** access to and control of resources**,** women’s situation relative to men**,** agency (control over their lives, environment), women’s human rights and gender equality [17–22]. There is no standard definition or measure of women’s status, however, common latent terms that can be implied from the discourse of women’s status are option, power, choice, control [17, 18]. The United Nations Commission on the Status of Women defines ‘women's status’ as the legal, economic, political, and social conditions of women and their relationship to society [23]; while empowerment is a related term focusing on women’s degree of control over their own lives and environments and over the lives of those in their care, such as their children [21]. Gender inequality in varying degrees and in different spheres of life feeds directly into the status accorded to women in the society. Thus, empowering women can foster gender equality, ultimately improving their status [21, 24]. Women’s status has evolved to include specific rights of women since the adoption of the United Nations Convention on Elimination of all Forms of Violence Against Women (CEDAW) [23, 25]. The terms, concepts and definitions of women’s status in the literature imply the various dimensions of women’s status, composed of several different and often interdependent variables [20, 23].

A study using the 2007 Bangladesh Demographic Health Survey found that an autonomy index (11 items related to decision-making, attitudes about partner violence, and freedom of movement) was associated with reduced risk of IPV [26]. In another study, the effect of women's status on violence was found to be context-specific. Indices of women’s autonomy/mobility, decision-making power, and control of resources were positively associated with past-year physical violence in a culturally conservative area of Bangladesh [27]. Women’s higher autonomy was a stronger protective factor against the risk of domestic violence in the southern state of Tamil Nadu than in the culturally conservative northern state of Uttar Pradesh [28].

Furthermore, studies have identified other factors that are protective against or put women at risk of IPV. Some of these factors include age, employment, educational attainment, witnessing mother being beaten during childhood, family type, duration of union, participation in household decision-making, partner’s alcohol use, partner’s employment status relative to woman, educational level differences between partner and woman, attitudes towards wife-beating among men and women, male right to discipline or control female behavior, among others [4, 29–32].

Over and above individual-level factors, contextual factors such as gender-related sociocultural norms at the community level may play a significant role in influencing the risk of IPV [16, 33, 34]. These norms are shared expectations of how men and women should behave, and they are highly influential in shaping individual behaviour [35, 36]. Deviations from these expected behaviours can attract shaming, sanctions, or disapproval by others [5, 35]. Examples are norms that men have the right to correct or discipline their wives and control their behavior [21, 37, 38]. This is seen in data from many countries, also showing both men and women justifying wife-beating under certain circumstances [4, 39–41]. Although justification of wife-beating is highly predictive of IPV, men’s attitude may be a stronger predictor than women’s attitude [5, 42]. Regressive community norms about women’s status and roles may not only influence the likelihood of IPV but may also reverse or mute the relationship between women's status and IPV [43]. In Nigeria, permissive social norms (husband’s right to beat his wife) at the state level appeared to significantly increase the odds of spousal violence [44]. Recently, another study in Nigeria showed that women’s engagement in cash work was positively associated with physical and sexual IPV victimisation. Residing in localities with greater male approval of wife-beating increased the positive association between engagement in cash work and IPV [30]. In one Indian study, the protective effect of higher education against IPV was muted in communities that approved of IPV [34].

This study goes beyond examining individual-level factors as separate indicators, to create a women’s status index based on the indicators. Also, we analyse IPV as consisting of physical, sexual, and emotional violence, as the different forms frequently overlap in occurrence [45]. The objectives were to 1) report the prevalence of different forms of IPV in Nigeria; 2) determine the association between women's status and IPV, controlling for other individual characteristics; 3) explore the differences in IPV across communities; 4) assess the contribution of individual- and community-level characteristics to community-level differences; and 5) examine the moderating influence of community social norms on the association between women’s status and IPV.

## Methods

### Study design and data collection

This was a cross-sectional study that used data from the population-based 2013 Nigerian Demographic and Heath Survey (DHS). The DHS collected data from February – June 2013, via a stratified three-stage cluster sample design using a sampling frame containing the list of enumeration areas prepared for the 2006 Population Census of the Federal Republic of Nigeria [6]. Contiguous enumeration areas were joined to make a DHS cluster (primary sampling unit [PSU] representing one community each). The sampling yielded 904 PSU and 40,320 households from rural and urban areas [6].

However, we used only 896 PSUs in our analysis, as these were the ones covered for the IPV data. Each PSU had approximately 42 observations. A minimum of 30 observations per group, and 30 groups at the second level of the analysis is recommended [46, 47]. For cross level interactions, a minimum of 20 observations per group and a minimum of 50 groups is recommended [48], while 200 groups with minimum 20 observation per group is recommended if the slope variance is estimated [49]. Increasing the number of PSUs will yield more precise estimates of community effects than increasing the number of people within the PSUs [47].

Trained DHS field interviewers speaking the same language as respondents collected data using questionnaires by face-to-face interviews. Women aged 15–49 years in each household were eligible for interview. Also, a subsample of one eligible woman per household was randomly selected to be asked additional questions regarding domestic violence. Where there was more than one eligible woman in a household, the DHS used the Kish grid to select one woman [6]. Furthermore, in every second household, all men aged 15–49 years who were either permanent residents of the households or visitors present in the households on the night before the survey were eligible to be interviewed. Men were interviewed using a questionnaire that was similar to, but shorter than the women’s questionnaire. Details of the survey design and sampling procedure are discussed elsewhere [6].

Of the 39,948 women who participated in the survey, 27,749 (69.4%) were randomly selected to be interviewed for the domestic violence module. Given that the present study focused on IPV, 6745 (24.3%) women were excluded, as they had never been in a relationship. Thus, data for this study were based on 21,004 ever-partnered women. IPV was assessed in the DHS based on a modified, shortened, and previously validated version of the Conflict Tactics Scale (CTS) [50]. In total, 202 (0.96%) women were excluded due to missing data of one or more variables, bringing the final number to 20,802 women in 896 PSU. A total of 17,359 men were interviewed, however only data from 17,194 men were analysed in this study due to missing data of 165 (0.95%).

### Study setting

The presence of 374 ethnic groups in Nigeria’s 36 states mean that cultural practices and gender norms differ [51, 52]. The Tiv-speaking people of North Central Nigeria believe wife-beating is a sign of affection and love [53]. Among the Igbos in South Eastern Nigeria societal privileges such as traditional titles, lands, wealth and decision-making are male-centered, and exclude women [51, 52]. Marriage customs in the largely patriarchal society of Nigeria involves payment of bride price. This practice often gives men an excuse to lay claims to ownership of their wives [7, 52].

In 1984, Nigeria became a signatory to the CEDAW, and ratified it in 1985 [6], but this has done little to protect women from discrimination and violence due to the long and laborious process of enforcing it [54–56]. The Nigerian criminal code makes provision for punishing unlawful and indecent assault on women, girls and men; three years imprisonment for assault on men, while assault on women and girls is punishable with two years imprisonment [57]. There seems to be a contradiction, however, as the Penal code, which governs the states in Northern Nigeria, allows husbands to “correct their wives using physical punishment, so long as the woman is not seriously harmed”. Furthermore, cases of domestic violence in Nigeria are hardly ever brought to trial as law enforcement agents consider domestic violence to be family affairs which should be resolved within the family. Particularly in rural areas, police do not respond if they consider the cases to be within cultural norms [58]. It is challenging to harmonize legislation and eradicate discriminatory measures due to the concurrent implementation of civil, customary and religious laws which sometimes contradict each other [59].

### Operationalisation of variables

#### Outcome variable

IPV as the outcome of interest was measured as physical violence, sexual violence, and emotional violence. Questions included experiences of one or several of the following acts of abuse by a current or former partner*:*

- *Physical violence: i)* pushing, shaking, or throwing something at her; *ii)* slapping her; *iii)* twisting her arm or pulling her hair; *iv)* punching her with his fist or hitting her with something harmful; *v*) kicking, dragging, or beating her; *vi)* choking or burning her on purpose; and *vii*) threatening or attacking her with a weapon (e.g., gun or knife).
- *Sexual violence: viii)* forced sexual intercourse; *ix)* physically forcing her to perform any other sexual act when undesired; and *x)* forcing her with threats to perform sexual acts when undesired.
- *Emotional violence: xi)* humiliating her in public; *xii)* threatening to hurt or harm someone close to her; and *xiii)* insulting or making her feel bad about herself.

Physical violence had a Cronbach’s alpha of 0.82; sexual violence by items *viii–x*, α = 0.84; emotional violence by items, α = 0.74; and any physical, sexual, or emotional violence, α = 0.87, indicating overall good test performance of the interview questions. A respondent was considered to have experienced IPV if she answered yes to at least one act of any of the forms of violence (physical, sexual, or emotional).

#### Exposure variables

##### Individual-level factors

Women's status –Women's status/empowerment encompasses several dimensions of a woman’s life – socio-cultural, economic, familial/interpersonal, political, legal -, at various levels – individual, family/household, community and the larger society. Practically, it is not so easy to separate these dimensions, as they may overlap considerably [18, 20]; thus, for the present study variables were selected that function as proxies for different dimensions [18, 20, 21]. Eleven single items were used to create a women's status index via principal component factor analysis. The Kaiser–Meyer–Olkin Measure of Sampling Adequacy (KMO) was 0.81, indicating that the variables used in creating the index were adequate for principal component analysis.

*Employment status and earnings:* Respondents were asked whether they were working, to which they responded ‘yes’ or ‘no’. Those who answered ‘yes’ were further asked what kind of earnings they got from their jobs; responses were ‘no earnings’, ‘cash earnings’, and ‘earnings in kind’.

*Control of income:* Respondents were asked who decided on how their earnings were used. Responses were categorised as ‘no earnings’, ‘has no control over her earnings’, and ‘decides solely or jointly with her partner’.

*Education* was categorised as illiterate, primary, secondary, or higher. *Media exposure:* An ordinal variable created from responses to three individual questions about how often a respondent read newspapers, listened to radio, or watched television. Responses were categorised as ‘no exposure’, ‘less than weekly exposure’, or ‘weekly exposure or more’.

*Age at first marriage/cohabitation* defined as median age in years when women aged 15–49 first married or lived with consensual partner [21]. This variable was categorised into four age groups: ‘less than 18’, ‘18–24’, ‘25–31’, and ‘more than 31’.

*Participation in household decision-making* measures women’s participation in the following items: *who decides on the woman’s healthcare, who decides on large household purchases, and who decides on visits to relatives.* For each item, a woman participates in decision-making when she alone or jointly with someone makes the decision. Responses are categorized as “no participation” or “participation”. These items reflect the degree of decision-making that a woman can exercise in areas that affect her own life and environment [21].

Eleven items were analysed for the index, from which 11 factors were generated; each factor, corresponding to one item. The Kaiser criterion, a rule of thumb, was used to determine the number of factors to be retained. Based on this criterion, factors with eigenvalues greater than 1 were retained, leading to three factors being retained [60]. These three factors explained about 72% of the total variability in the original 11 items. Some scholars have argued that the Kaiser criterion could lead to overestimation in the number of factors extracted [61], thus, the scree plot (see Additional file 1) was also used in conjunction with the Kaiser criterion to determine the number of factors to retain. The ideal pattern of the scree plot is a steep curve followed by a bend (elbow), which then begins to flatten out. The number of factors to be retained is the data points above the bend [60].

Furthermore, we examined the loadings of the three factors retained, on each of the original 11 items used in the analysis (see Additional file 2). The factor loading for a variable is a measure of how much the variable contributes to the factor; thus, high factor loading scores indicate that the dimensions of the factors are better accounted for by the variables [60]. A general rule is that for larger sample size, smaller loadings are allowed for a factor to be considered significant [62]. For a sample size of at least 300, a rotated factor loading of 0.32 is needed for the factor to be considered statistically meaningful [60, 61]. Items 1–3 load highest on factor 1. These items representing employment, income and control of income, correspond to the economic dimension of women’s status. Items 4–8 loads highest on factor 2, and represent exposure to newspaper, radio and television, education level and age at first cohabitation/marriage. These correspond to the social dimension of women’s status. Factor 3 has the highest correlation with items 9–11, which represent participation in household decision-making and correspond to the familial dimension. The uniqueness is the proportion of variation in an item not explained by a factor. Values more than 0.6 are usually considered high, which means that variable is not well explained by the factors [60].

The analysis predicted an index score for each woman, which was categorized into tertiles – low, middle and high status.

##### Covariates

Socio-demographic characteristics: *i)* age (four categories, 15 to 49 years), *ii)* place of residence (urban or rural), and *iii)* household wealth (categorised by the DHS into quintiles). Details of the wealth quintiles creation can be found elsewhere [63].

Attitude towards wife-beating – a categorical ‘yes’ or ‘no’ variable was created from responses to five scenarios: *if she goes out without telling him; if she neglects the children; if she argues with him; if she refuses to have sex with him; and if she burns the food.* An answer of ‘yes’ to at least one scenario meant the respondent justified wife-beating and was coded as 1, while an answer of no in all scenarios meant the respondent did not justify wife-beating and was coded as 0. Cronbach’s alpha of reliability calculated in this study for the items was 0.89. The above-mentioned five scenarios were chosen based on prevailing socio-cultural gender norms relations (6,40). Patriarchal societies are characterised by power relations and men’s authority over women. In these societies, women are expected to care for children, prepare food properly, keep the house clean, attend to husband’s sexual need, obtain husband’s permission before going out, be submissive to husband. Transgression of these expectations could be a trigger for wife-beating in a bid to discipline the woman [14, 15, 52].

Partner’s controlling behaviour – a binary ‘yes’ or ‘no’ variable – was derived from responses to five items: *jealous if she talks with other men; accuses her of unfaithfulness; does not permit her to meet her friends; tries to limit her contact with family; and insists on knowing where she is always.* Women who responded ‘yes’ to one or more questions were categorised as having a partner/husband with control issues. Women who responded ‘no’ to all the questions were categorised as not having a partner with control issues. This was based on only women’s responses. Cronbach’s alpha for this item was 0.90. The DHS included these series of questions to assess the degree of control exercised by a husband/partner over the respondent. An important early warning sign of violence in a relationship is control and close monitoring of women by their husbands/partners [6].

##### Contextual factors

*i) Community norms about wife-beating* was created by aggregating responses from men in each community. Men were asked if wife-beating was justified in the following scenarios: *if she goes out without telling him; if she neglects the children; if she argues with him; if she refuses to have sex with him; and if she burns the food.* Communities were categorised as ‘does not justify wife-beating’ if the proportion of men was 0% and ‘justifies wife-beating’ if the proportion of men that justified wife-beating was above 0%. *ii) Control over female behaviour* was created by aggregating women’s responses about their partner’s controlling behaviour in each community. Communities were grouped into tertiles of low, moderate, and high levels of control over female behaviour.

##### Statistical analyses

Descriptive analysis was conducted to present the proportion of women who experienced any IPV for each category in the explanatory variables. To compensate for non-response rates and women’s unequal selection probability, sampling weights (DHS domestic violence weights) were introduced in the descriptive statistics, and the results of the descriptive analysis were presented as numbers and weighted percentages. Bivariate analysis was performed via simple logistic regression to assess the association between individual women characteristics and IPV. The significance level was set at *p*-value = 0.05. Due to the hierarchical structure of the data, where individuals are nested within PSU (communities), a multiple multilevel logistic model [64, 65] with two levels (individual and community) was fitted to assess the effects of measured individual- and community-level (fixed effect) characteristics on women’s experience of IPV, and to estimate the extent of variations across communities (random effects).

Six models were fitted: A null model with no explanatory variables was used to show variation across communities and to justify the use of multilevel analyses. Model 2 contained only individual variables, showing random intercepts and fixed slopes. This model studied the association between women's status and IPV, adjusting for other potential confounders in the association and showed how much of the variation in IPV across communities was explained by individual-level factors. Model 3 was like model 2, but also contained community variables to show measures of association and to quantify how much community-level factors explained the IPV over and above individual-level factors. Model 4 was a random intercept random slope model, with individual variables only. We assumed that the effect of women status on IPV might be different from one community to another. In that case, the slope of the association between women’s status and IPV would vary from one community to another and community disparities become a function of individual women's status. Model 5 was like model 4 but included community-level variables. Each community had its own coefficient for the association between individual women’s status and IPV exposure. The random slope analyses provided information about whether the association between women status and IPV differed across communities to ultimately justify the examination of cross-level interaction. Model 6 was a full model that included a term for interaction between individual women’s status and men’s justification of IPV at the community level. We tested for only one cross level interaction.

##### Fixed effects (measures of association)

The results were expressed as odds ratios (OR) with 95% confidence intervals (CI). Statistical significance was determined at *p*-value < 0.05.

##### Random effects (measures of variation)

We calculated the second-level variance (variation between communities) regarding the prevalence of IPV (i.e., the intercepts in the multilevel logistic regression) and the second-level variance regarding the association between women status and experience of IPV (i.e., the slope variance in the multilevel regression). The slope variance tells us how each community’s coefficient for the association between women's status and IPV deviates from the population average. We also calculated the covariance between intercept and slope residuals. The covariance gives information about whether the association between individual women status and IPV depends on the community norms regarding IPV in the different communities (i.e., cross-level interaction). We also applied the intra-class correlation (ICC) and median odds ratio (MOR) to test the hypothesised phenomenon that individuals living in the same community shared a similar probability of experiencing IPV, after adjusting for the individual characteristics studied. The ICC gives us the proportion of the total variation at the community level, while the MOR expresses the community variance in the OR scale. If the MOR is equal to 1 (no community-level variation), there is no difference between the communities regarding IPV. The higher the MOR, the more important the contextual effects for understanding the individual probability of experiencing IPV.

The model fit was analysed using deviance information criterion (DIC) as a measure of how well our different models fitted the data. A lower value in DIC indicates a better fit of the model [66]. Parameters in the model were estimated using the mean–variance adaptive Gauss–Hermite. The Stata Version 14.1 (Stata Corp. Inc., TX, USA) software package was used for the analyses.

##### Ethical consideration

The survey procedure and instruments used in the DHS had already received ethical approval from the National Health Research Ethics Committee of the Federal Ministry of Health of Nigeria and the Ethics Committee of the Opinion Research Corporation Macro International, Inc. (ORC Macro Inc., Calverton, MD, USA). In line with WHO recommendation, only one woman per household was interviewed so that no one else in the household knew which issues were discussed. Interviewers reiterated informed consent immediately prior to administering domestic violence questions. Care was taken by interviewers to ensure privacy; where this was not possible, the interview was not conducted, or it was terminated if privacy was breached [6]. Permission to use the DHS data in the present study was obtained from ORC Macro Inc. The dataset does not contain any individual identifiers that would make it possible to track any participant.

## Results

### Characteristics of the study population

Table 1 shows characteristics of the study sample by experience of IPV, with bivariate association between the lifetime experience of IPV and individual-level exposure variables. The average age of women in the sample was 31 years. Thirty-four percent of women were of middle status, and 63% lived in rural areas. Twenty-two percent of the sample lived in the poorest households. Eighty-seven percent of the sample lived in communities where men justified wife-beating. Experience of IPV was lowest among the oldest age group, but highest among women aged 25–34 years. Also, the frequency of IPV was higher among women living in rural areas, those belonging to the middle wealth quintile. There was regional variation in the frequency of IPV; the proportion of women that reported experience of IPV was highest in the North East (21.9%) and lowest in the South East (11.8%).

**Table 1:** Characteristics of the study sample and bivariate analysis by experience of any intimate partner violence

	Never experienced IPV	Experienced IPV	Total
	N (%)	N (%)	N (%)
Women's status	*p* < 0.001		
Low	5168 (32.7)	1764 (33.5)	6932 (32.8)
Middle	5138 (33.8)	1815 (35.2)	6953 (34.1)
High	5296 (33.6)	1621 (31.3)	6917 (33.0)
Age groups	*p* < 0.001		
15–24	4046 (25.1)	1071 (19.9)	5117 (23.9)
25–34	6229 (37.7)	2253 (40.4)	8482 (38.3)
35–44	3870 (26.3)	1446 (29.9)	5316 (27.2)
45–49	1591 (10.9)	454 (9.7)	2045 (10.6)
Residence	*p* < 0.001		
Urban	5547 (35.3)	1976 (41.1)	7523 (36.7)
Rural	10,055 (64.7)	3224 (58.9)	13,279 (63.3)
Wealth level	*p* < 0.001		
Poorest	3455 (25.3)	764 (14.1)	4219 (22.7)
Poorer	3283 (21.3)	1108 (21.5)	4391 (21.4)
Middle	2819 (16.9)	1157 (21.4)	3976 (18.0)
Richer	2948 (17.5)	1144 (21.0)	4092 (18.3)
Richest	3097 (19.0)	1027 (22.1)	4124 (19.7)
Region	*p* < 0.001		
North Central	2384 (12.6)	943 (17.9)	3327 (13.8)
North East	2396 (14.8)	1427 (21.9)	3823 (16.5)
North West	5506 (42.5)	514 (17.2)	6020 (36.5)
South East	1296 (7.3)	589 (11.8)	1885 (8.4)
South South	1743 (8.3)	958 (14.5)	2701 (9.8)
South West	2411 (14.6)	793 (16.8)	3204 (15.1)
Partner’s controlling behaviour	*p* < 0.001		
No controlling behaviour	6584 (41.6)	832 (17.2)	7416 (35.9)
Has controlling behaviour	9018 (58.4)	4368 (82.8)	13,386 (64.1)
Woman’s attitude to wife-beating	*p* < 0.001		
Does not justify wife-beating	10,030 (65.8)	2725 (53.4)	12,755 (62.8)
Justifies wife-beating	5572 (34.2)	2475 (46.6)	8047 (37.2)
Community level male justification of wife-beating	*p* < 0.001		
No justification	2023 (14.2)	378 (6.7)	2401 (12.5)
Justification	13,579 (85.8)	4822 (93.3)	18,401 (87.6)
Community level control of female behaviour	*p* < 0.001		
Low	5681 (35.3)	998 (19.6)	6679 (31.6)
Moderate	4804 (29.4)	1861 (39.1)	6665 (31.7)
High	5117 (35.3)	2341 (41.4)	7458 (36.8)
TOTAL	15,602 (76.4)	5200 (23.6)	20,802 (100)

Significance level- α < 0.05; Percentages (%) are weighted. *N* = 20,802

The frequency of IPV increased in younger women aged 15–24, peaked at 40.4% among women aged 25–34 years and declined to 9.7% among women aged 45–49 years. Sixty-four percent of women in the sample had a partner who exhibited controlling behaviour, and 62% did not justify wife-beating. Women whose partners exhibited controlling behavior were more likely to have experienced IPV. However, women who did not justify wife-beating (53%) reported experience of IPV more frequently than those who justified (47%) wife-beating. Results from Table 1 also show that women who reported experience of IPV differed statistically significantly from women who reported no experience of IPV, in women status and other characteristics examined. Baseline characteristics of the men interviewed are shown in Additional file 3.

### Prevalence of IPV among women

**Fig. 1:**
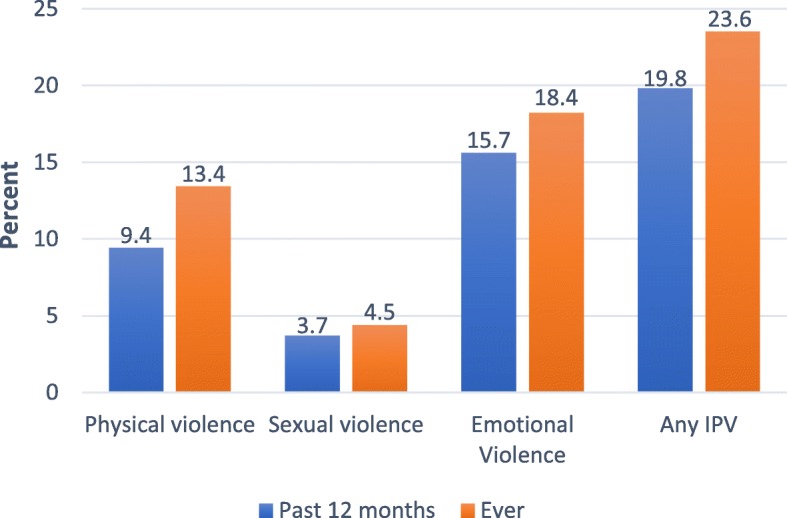
Proportion of respondents who reported experience of any Intimate Partner Violence (IPV) and the different forms of IPV

Figure 1 shows the proportion of women who reported any IPV and the different forms of IPV. Almost one in four women reported experience of any IPV ever (23.6%), while one in five (20%) reported experience of any IPV in the 12 months preceding the survey. Of the three forms of violence, emotional violence had the highest frequency (18% ever and 16% in the 12 months preceding the survey).

### Experience of different forms of IPV, singly or in combination

**Fig. 2:**
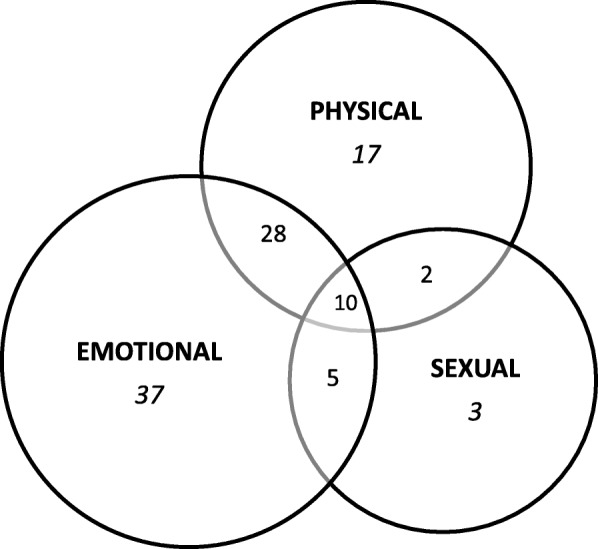
Overlap between the forms of intimate partner violence among women who reported experience of IPV

Women reported experiencing the different forms of IPV either singly or in combination. Figure 2 below shows the overlap between the different forms of violence among women who reported experience of one or more forms of IPV. The figures represent the proportion of abused women (*n* = 5224) who reported experience of a specific form or combinations of different forms of violence. For example, about 10% of women who had experienced any IPV reported experience of all three form of IPV. Thirty-seven  percent of women who had experienced IPV reported experience of emotional violence alone.

## Multilevel analyses

The results of multilevel logistic regression analysis are shown for experience of any IPV in Table 2. The data were examined to see if the decision to assess random effects at the community levels was justified based on the results of the random intercept-only model (null model). The null model showed a community level variance of 2.04 with a standard error (SE) of 0.13, which was significant, as the variance is greater than two times the SE. The caterpillar plot (see Additional file 4) shows the estimated residuals for all 896 communities in the sample. For a substantial number of communities, the 95% CI does not overlap the horizontal line at zero, indicating that IPV in these communities is significantly above average (above the zero line) or below average (below the zero line).

**Table 2:** Multilevel logistic regression of the association between experience of IPV and women’s status among ever-partnered women in Nigeria

Levels and variables		Models				
	1 Null model	2 Random intercept and fixed slope- Individual variables only	3 Random intercept and fixed slope- Individual and community variables	4 Random intercept and random slope- individual variables only	5 Random intercept and random slope- individual and community variables	6 Cross-level interaction
INDIVIDUAL LEVEL (L1)						
Women status						
Low		1	1	1	1	1
Middle		1.01 (0.89–1.13)	1.00 (0.89–1.12)	0.88 (0.77–1.02)	0.90 (0.78–1.04)	0.75 (0.51–1.11)
High		0.94 (0.82–1.07)	0.92 (0.80–1.05)	0.84 (0.72–0.98)	0.85 (0.73–1.00)	0.47 (0.32–0.71)
Age						
15–24		1	1	1	1	1
25–34		1.25 (1.12–1.40)	1.26 (1.12–1.41)	1.26 (1.15–1.44)	1.26 (1.15–1.44)	1.26 (1.12–1.42)
35–44		1.35 (1.19–1.54)	1.36 (1.20–1.55)	1.38 (1.25–1.62)	1.38 (1.26–1.63)	1.38 (1.21–1.58)
45–49		1.22 (1.02–1.45)	1.22 (1.03–1.46)	1.22 (1.07–1.52)	1.23 (1.08–1.53)	1.23 (1.03–1.47)
Wealth quintile						
Poorest		1	1	1	1	1
Poorer		1.25 (1.06–1.47)	1.26 (1.07–1.49)	1.26 (1.06–1.48)	1.27 (1.08–1.50)	1.27 (1.07–1.50)
Middle		1.39 (1.15–1.69)	1.41 (1.17–1.71)	1.42 (1.17–1.73)	1.43 (1.18–1.74)	1.43 (1.18–1.74)
Richer		1.36 (1.09–1.69)	1.40 (1.12–1.74)	1.39 (1.12–1.74)	1.42 (1.14–1.77)	1.42 (1.14–1.76)
Richest		1.18 (0.92–1.51)	1.22 (0.95–1.57)	1.20 (0.94–1.55)	1.23 (0.96–1.59)	1.24 (0.96–1.59)
Nature of union						
Monogamous		1	1	1	1	1
Polygamous		1.19 (1.07–1.33)	1.19 (1.07–1.32)	1.19 (1.07–1.32)	1.18 (1.06–1.32)	1.18 (1.06–1.32)
Place of residence						
Urban		1	1	1	1	1
Rural		0.98 (0.80–1.21)	0.95 (0.77–1.16)	1.02 (0.83–1.25)	0.97 (0.79–1.19)	0.98 (0.80–1.20)
Woman’s attitude to IPV						
Does not justify wife-beating		1	1	1	1	1
Justifies wife-beating		1.32 (1.20–1.45)	1.30 (1.18–1.43)	1.33 (1.21–1.47)	1.31 (1.19–1.44)	1.31 (1.19–1.44)
Witnessed mother being beaten						
No		1	1	1	1	1
Yes		2.44 (2.15–2.78)	2.44 (2.15–2.78)	2.48 (2.17–2.82)	2.47 (2.17–2.82)	2.48 (2.17–2.82)
Partner’s alcohol use						
Does not drink		1	1	1	1	1
Never gets drunk		2.09 (1.71–2.56)	2.15 (1.76–2.63)	2.15 (1.76–2.64)	2.20 (1.80–2.70)	2.20 (1.79–2.70)
Gets drunk sometimes		2.42 (2.13–2.75)	2.41 (2.12–2.74)	2.48 (2.17–2.82)	2.46 (2.16–2.81)	2.47 (2.16–2.81)
Gets drunk often		5.89 (4.63–7.50)	5.89 (4.62–7.49)	6.09 (4.77–7.78)	6.08 (4.76–7.77)	6.09 (4.77–7.78)
Partner’s controlling behaviour						
None		1	1	1	1	1
Yes		4.01 (3.61–4.47)	3.83 (3.43–4.26)	4.05 (3.63–4.52)	3.87 (3.47–4.32)	3.87 (3.46–4.32)
Partner’s education level						
Tertiary		1	1	1	1	1
Secondary		1.24 (1.08–1.43)	1.24 (1.07–1.42)	1.24 (1.08–1.43)	1.24 (1.07–1.43)	1.24 (1.07–1.43)
Primary		1.41 (1.19–1.68)	1.41 (1.19–1.68)	1.40 (1.18–1.67)	1.41 (1.18–1.67)	1.41 (1.18–1.67)
No education		1.19 (0.88–1.60)	1.17 (0.87–1.58)	1.16 (0.86–1.58)	1.15 (0.85–1.56)	1.15 (0.85–1.55)
Education difference between partners						
Both partners educated equally		1	1	1	1	1
Partner more educated than woman		1.12 (0.99–1.26)	1.11 (0.98–1.25)	1.11 (0.98–1.25)	1.10 (0.98–1.24)	1.10 (0.97–1.25)
Woman more educated than partner		1.11 (0.95–1.28)	1.11 (0.95–1.29)	1.11 (0.95–1.29)	1.11 (0.95–1.30)	1.11 (0.95–1.30)
Both partners are not educated		0.72 (0.54–0.95)	0.72 (0.54–0.95)	0.71 (0.53–0.94)	0.71 (0.54–0.94)	0.71 (0.53–0.94)
Income difference between partners						
Both partners earn equally		1	1	1	1	1
Partner earns more than woman		1.24 (0.99–1.54)	1.23 (0.99–1.53)	1.23 (0.98–1.53)	1.22 (0.97–1.52)	1.23 (0.98–1.53)
Woman earns more than partner		1.69 (1.24–2.31)	1.67 (1.23–2.28)	1.70 (1.24–2.33)	1.68 (1.23–2.30)	1.67 (1.22–2.29)
Both partners do not earn		1.20 (0.94–1.52)	1.16 (0.91–1.48)	1.16 (0.91–1.49)	1.14 (0.89–1.46)	1.15 (0.90–1.47)
COMMUNITY LEVEL (L2)						
Men’s attitude to IPV						
Does not justify wife-beating			1		1	1
Justifies wife-beating			2.13 (1.58–2.87)		2.08 (1.54–2.81)	1.66 (1.17–2.35)
Control over female behaviour						
Low			1		1	1
Moderate			1.68 (1.34–2.09)		1.66 (1.33–2.07)	1.66 (1.33–2.07)
High			1.88 (1.49–2.36)		1.82 (1.44–2.30)	1.81 (1.44–2.29)
Cross-level interaction between women status (L1) and Men’s attitude to IPV (L2)						
Low status X Men’s attitude						1
Moderate status X Men’s attitude						1.21 (0.82–1.79)
High status X Men’s attitude						1.89 (1.26–2.83)
Variance components (SE)						
Intercept (L2) variance	2.04 (0.13)	1.45 (0.10)	1.34 (0.10)	1.17 (0.14)	1.18 (0.14)	1.14 (0.14)
Slope (L1)_1_ variance				0.39 (0.13)	0.40 (0.13)	0.39 (0.13)
Slope (L1)_2_ variance				0.28 (0.12)	0.29 (0.12)	0.25 (0.10)
Intercept-slope (L1)_1_ covariance				0.16 (0.11)	0.06 (0.11)	0.08 (0.11)
Intercept-slope (L1)_2_ covariance				0.11 (0.10)	.003 (0.10)	0.06 (0.10)
Slope (L1)_1_-slope (L1)_2_ covariance				0.28 (0.10)	0.30 (0.10)	0.28 (0.10)
General contextual effects						
ICC (%)	38.3	30.6	28.9	26.2	26.4	25.7
MOR	3.88	3.14	3.00	2.79	2.81	2.76
PCV (%)		28.9	7.6	12.7	0.85	3.4
Model Fit Statistics						
Deviance Information Criteria	19,823.92	15,817.98	15,761.46	15,783.44	15,732.48	15,722.33
Deviance change		4005.94	56.2	−21.98	50.96	10.15

L1: level 1. L2: level 2. *ICC* intra-class correlation coefficient, *MOR* median odds ratio, *PCV* proportional change in the variance, *DIC* deviance information criterion. These models were adjusted for age, wealth quintile, place of residence, women’s attitude to IPV, woman witnessed parental violence, partner’s alcohol use, partner’s controlling behaviour, partner’s education level, education difference between partners and income difference between partners

### Fixed effects – Specific individual observational effects

A single-level multivariate logistic regression (see Additional file 5) showed a significant negative association between higher women’s status and IPV, but the association was not significant for women of middle status. Across the multilevel models fitted, the higher a woman’s status, the lower her probability of experiencing IPV (Table 2). In model 6, women with high status had a 53% less chance (OR = 0.47; CI = 0.32–0.71) of experiencing IPV compared to women of low status. Although women of middle status also had a lower probability of experiencing IPV compared to women with the lowest status, the association was not significant. Age showed positive significant association with experience of IPV through all the models, peaking at 35–44 years, and declining in older years (45–49 years). The odds of IPV were higher for all wealth quintiles, but not uniformly. Partner’s controlling behaviour (OR = 3.87; CI = 3.46–4.32) and women’s attitude to IPV (OR = 1.31; CI = 1.19–1.44) also showed positive significant association with IPV. Women residing in rural areas had lower odds of experiencing IPV compared to their counterparts in urban areas. However, this association was not significant. Polygamous union, partner’s alcohol use, and witnessing mother being beaten during childhood all increased the odds of IPV. Partner’s education level and income difference between the woman and her partner and education difference showed positive association with IPV, except where both partners were not educated, which showed significant negative association with IPV.

Residing in a community where men justified wife-beating was positively associated with experience of IPV (OR = 1.66; CI = 1.17–2.35). Also, residing in a community where the level of control over women’s behaviour was high, increased a woman’s chance of experiencing IPV by 81%.

**Fig. 3:**
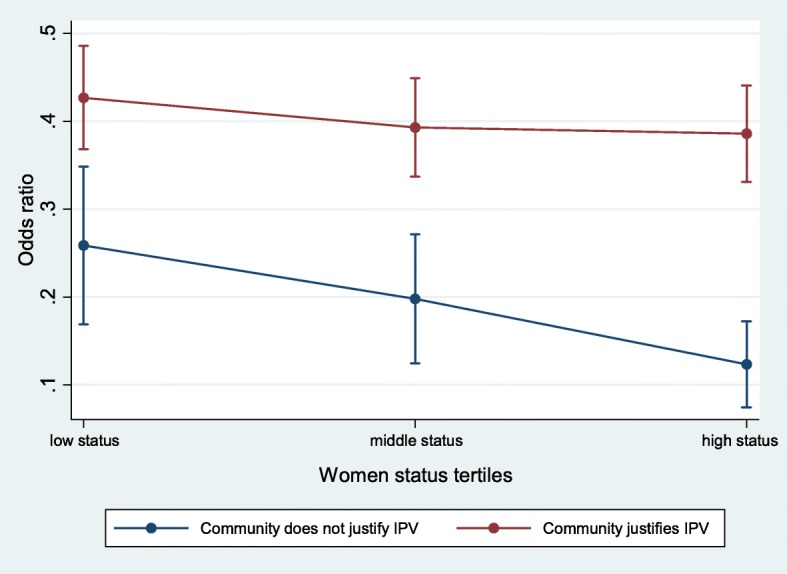
Fixed effect cross level interaction between men’s justification of IPV in the community and individual women’s status

A significant cross-level interaction can be seen in model 6 between men’s justification of IPV in the community and individual women’s status for women of high status (OR = 1.89; CI = 1.26–2.83), so the odds of IPV occurrence among women with high status was greater in communities where men justified IPV (Table 2). Figure 3 above illustrates the cross-level interaction.

### Random effects

Across the models, the inter-community variance of IPV decreased from 2.04 in model 1 to 1.14 in model 6 upon inclusion of additional explanatory variables through the models. The variance of 1.14 in model 6 indicates that there is still some residual variance between communities in experience of IPV after adjustment for individual and community characteristics. Inclusion of men’s justification of IPV and control over women’s behaviour in model 3 explained 7.6% of the community differences in IPV.

The ICC and MOR through the models were quite high, ranging from 38.3 to 25.7% and 3.88 to 2.76%, respectively. This indicated that the community is a relevant context for understanding a woman’s propensity of experiencing IPV. In the null model, the ICC indicated that 38.3% of the total variation in prevalence of IPV is attributable to between-community differences.

For multilevel logistic regression, within community variance is fixed, thus, the variance displayed in the regression results in Table 2, was between-community variance. The overall variance between communities as derived from the null model was 2.04, that is an ICC of 38.3%. This means that the between community variance contributed 38.2% to the overall variance of IPV in the population. With addition of variables at the individual and community levels in the model, the proportion of variance explained by the variables changed (see Additional file 6).

## Discussion

This study analysed the 2013 Nigerian DHS to examine the association between women’s status and IPV in the face of prevailing community social norms in Nigeria. Our study revealed that about one fourth of Nigerian women reported having ever experienced IPV. Within a multilevel framework informed by the ecological model [16], we found that IPV was influenced not only by individual characteristics such as women’s status, but also by contextual factors such as men’s attitude towards IPV at the community level, having adjusted for other covariates. In addition, the cross-level interaction between women’s status and men’s justification of IPV showed that the protective effect of high women’s status could be reversed if men accept violence against women in the community.

A study using the 2008 Nigerian DHS found that among ever-married women, 18.7% reported exposure to sexual or physical violence [44], while in our study 5224 (23.5%) ever-partnered women aged 15–49 reported experiencing at least one form of IPV (physical, sexual, emotional) at some point in their life. This prevalence was lower than the global lifetime prevalence of 30% from the WHO global and regional estimates of violence against women. Studies have reported lifetime IPV prevalence rates of 52.8% in Congo, 42% in Kenya, 27% in Malawi, 32% in Rwanda, 48% in Zambia, and 33% in Zimbabwe [5]. These estimates vary considerably, probably in part due to differences in definition and measurement of IPV. However, estimates also demonstrate the high prevalence of IPV as a public health concern.

We also found that experience of IPV varied across the regions of the country; this concurs with findings from other studies which have shown similar variations [8–11]. This variation may be due to differences and peculiarities in culture and traditions across regions, even though patriarchy is the norm countrywide. In our study, the proportions differed from previous reports; this may be due to the operationalization of the IPV variable; some studies studied only physical or only sexual violence and not all three forms of IPV as we did in our study.

Higher women’s status was negatively associated with IPV, although the association was not conclusive for women of middle status. Similarly, studies have found reduced risk of IPV in relation to higher women’s status [26–28]. Women with higher status may be able to decide when and whom to marry, and thus they will be less likely to enter an abusive relationship in the first place. It is likely that they would not justify wife-beating, and thus they are more likely to enter a relationship with a partner who holds similar views and not experience IPV. However, due to the cross-sectional nature of the study, we cannot be certain that women’s higher status preceded occurrence of IPV, or vice-versa. Thus, this will only hold true for women who acquired higher status before the occurrence of IPV.

The index we created included areas such as household decision-making, access to and control over economic resources, which also have been used in other studies [26–28]. However, indices created in other studies did not include education, literacy, media exposure or age at first cohabitation/marriage. Also, in our study, women’s status was protective at the highest level, but its effect was moderated by men’s acceptance of IPV at the community level. Similarly, in other studies, the impact of the indices on IPV was found to vary across cultures [27, 28].

Our results also showed that the prevalence of IPV was higher among women who did not justify wife-beating. This finding appears quite counterintuitive; perhaps these women are more at risk of IPV as they may act out their perceptions, thus attracting discipline from their partner who may consider them insubordinate [13, 15]. However, further analysis showed men’s justification of wife-beating increased a woman’s probability of experiencing IPV even more. Studies have shown that men’s views of IPV are stronger predictors of IPV than women’s views, as women’s perception may be more descriptive or injunctive rather than what they really think [5, 42]. Overall, a woman’s non-approval of IPV may not be enough to reduce her risk of experiencing it, as her status is also important.

The present study revealed an increase, a peak and then a decline in the odds of exposure to IPV as age increased. Younger women are more likely to have been in union for fewer years. Thus, the younger women may be more submissive and more accepting of her partner’s behaviour as she tries to win over her in-laws, establish her place in the family and try to make the union work. Even when she is experiencing IPV, she may not want to leave as she may be considered a failure, who was not able to keep her home. On the other hand, older women are more likely to have been in union for longer, may have become experienced in married life and know how to navigate situations with their partners to avoid conflict. Also, they may have acquired status by working, earning an income, and contributing to the family income and have some say in decision-making in the home. Studies have shown mixed results with regards to age and IPV. Some studies have shown that the risk of IPV declines with age [31], while others have shown variation with age [4, 67].

At the community level, men’s attitude towards IPV and control over women’s behaviour significantly increased the probability of IPV. Men’s justification of wife-beating in one or more situations has been found to strongly predict IPV, even more than women’s justification of IPV [5, 33, 42]. Previous studies in Nigeria have also shown that justification of wife-beating by both women and men increased the probability of IPV [30, 39, 44]. Also, men’s control over female behavior have been shown to increase the likelihood of IPV [16, 29, 44]. However, in our study, men’s acceptance of wife-beating at the community level also reversed the protective effect of higher status on the likelihood of IPV. This interaction, although small, underscores the importance of contextual factors in IPV occurrence. Even if a woman had high status, community norms among men tend to override the effect of her individual status. Men would readily go with the norm that expects them to be in control of their home affairs and discipline their partners when the need arises, since prevailing norms influence behavior and non-compliance with such norms can attract consequences [5, 16, 36]. Therefore, if individual women’s status was to be improved, a woman cannot realise the full benefits if there is no change in men’s attitudes towards intimate partner violence, particularly at the community level. A study showed that the protective effect of education against IPV was muted by community norms that approved of IPV [34]. Some studies have found that the protective effect of women’s status against IPV is absent in culturally conservative contexts [27, 28].

### Strengths and limitations

This study utilised nationally representative data with a large sample size, which strengthens the external validity and generalizability of the study. The use of a two-level analysis – individual-level and aggregated responses at the ‘community’ level - allowed for simultaneous examination of individual and contextual factors. Also, this study went beyond examining single factors related to women’s status, to construct a multi-dimensional women’s status index that was a used as the primary exposure variable. Furthermore, it contributes to efforts in exploring the interaction between socio-cultural IPV norms and individual-level characteristics in studies about IPV in Nigeria.

This study had some limitations that need to be considered when interpreting the results. The cross-sectional design of this study precludes us from drawing causal inference from the results obtained. Also, we cannot tease out the time-order of attitude towards wife beating from IPV experience. However, our aim was not to study the mechanism and causality. The method applied in this study can be used to disentangle contextual effects from individual effects, but since we estimated community-level factors based on individual responses, associations with IPV may be partly or wholly due to individual characteristics. Aggregating individual level responses to community level might lead to the ecological fallacy, when inference made about the association of individual level variables is based on the observed association of parallel group level variables [68]. However, collecting such information (i.e. community IPV norms among men) from the entire population is practically impossible, thus, aggregating individual responses is not only acceptable, but also the only way to address the importance of these issues. Similarly, other studies have aggregated individual responses to create community level variables [30, 34].

Only eight PSUs were not included in the analysis and this will probably not lead to selection bias. However, we do recognise that selection bias could arise if included PSUs are not representative of the population, and the association of the community level variable with the outcome in the selected communities is different from the association in the total population of PSUs [68].

The DHS survey teams were trained to ensure privacy and safety, and the interview instrument used was designed to enhance disclosure [6]. However, there is the possibility that the prevalence of IPV against women was underestimated in this population due to underreporting. This is a problem in many studies; it is not possible to control for such limitations. Further qualitative studies may help to better understand the extent of the problem. Finally, the women status index created has fewer dimensions than what is found in the literature [18, 20, 23]. We focused on these dimensions based on the available data; further studies are needed to broaden our understanding of women’s status. Further research should take these limitations into consideration.

## Conclusions

This study explored the negative influence of community IPV norms among men on the association between women’s status and IPV. Our findings underscore the importance of community socio-cultural norms and call for adoption of community-wide approaches aimed at changing norms alongside improvement of women's status. In designing prevention strategies, planners should keep in mind the complex dynamics between socio-cultural norms and individual factors. However, it is important to monitor and evaluate such prevention strategies adequately to provide evidence of the effectiveness of such programs.

## Additional files

Additional file 1: **Figure S1.** Scree plot of factors derived from the principal component factor analysis. (DOCX 16 kb)

Additional file 2: **Table S1.** Factor loadings of retained factors on the items analysed and proportions of variability not explained. (DOCX 14 kb)

Additional file 3: **Table S2.** Baseline characteristics of the men interviewed. (DOCX 20 kb)

Additional file 4: **Figure S2.** Caterpillar plot of residuals for null model, ranking communities by women’s reported experience of IPV. (DOCX 22 kb)

Additional file 5: **Table S3.** Single level logistic regression of the association between IPV, women’s status and covariates among ever-partnered women in Nigeria (DOCX 18 kb)

Additional file 6: **Figure S3.** Contribution of individual- and community-level to the intra-class correlation (ICC). (DOCX 27 kb)

## Abbreviations

CEDAW: Commission for the Elimination of Discrimination against women
CI: Confidence Interval
CTS: Conflict Tactics Scale
DHS: Demographic and Health Survey
DIC: Deviance Information Criteria
ICC: Intraclass Correlation
IPV: Intimate Partner Violence
KMO: Kaiser Meyer Olkin
L1: Level 1
L2: Level 2
LR: Likelihood Ratio
MOR: Median Odds Ratio
OR: Odds Ratio
PCV: Proportional Change in Variation
PSU: Primary Sampling Unit
SE: Standard Error
STIs: Sexually Transmitted Infections
WHO: World Health Organization
