# Supplementary material for: Intimate partner violence against women in Nigeria: a multilevel study investigating the effect of women’s status and community norms
Source: BMC Womens Health. 2018 Aug 9;18:136. doi: 10.1186/s12905-018-0628-7 (PMC6085661; doi:10.1186/s12905-018-0628-7)
Supplement: Supplementary file 1 — Figure S1. Scree plot of factors derived from the principal component factor analysis. (DOCX 16 kb) [file 12905_2018_628_MOESM1_ESM.docx]

Figure S1: Scree plot of factors derived from the principal component factor analysis.
