# Supplementary material for: Intimate partner violence against women in Nigeria: a multilevel study investigating the effect of women’s status and community norms
Source: BMC Womens Health. 2018 Aug 9;18:136. doi: 10.1186/s12905-018-0628-7 (PMC6085661; doi:10.1186/s12905-018-0628-7)
Supplement: Supplementary file 2 — Table S1. Factor loadings of retained factors on the items analysed and proportions of variability not explained. (DOCX 14 kb) [file 12905_2018_628_MOESM2_ESM.docx]

Table S1: Factor loadings of retained factors on the items analysed and proportions of variability not explained.

| Variable | Factor 1 | Factor 2 | Factor 3 | Uniqueness |
| --- | --- | --- | --- | --- |
| Item 1 | 0.9747 |  |  | 0.0363 |
| Item 2 | 0.9274 |  |  | 0.1176 |
| Item 3 | 0.9580 |  |  | 0.0629 |
| Item 4 |  | 0.7159 |  | 0.4802 |
| Item 5 |  | 0.6839 |  | 0.5071 |
| Item 6 |  | 0.7796 |  | 0.3467 |
| Item 7 |  | 0.7884 |  | 0.2998 |
| Item 8 |  | 0.5632 |  | 0.6081 |
| Item 9 |  |  | 0.8932 | 0.1642 |
| Item 10 |  |  | 0.8710 | 0.1926 |
| Item 11 |  |  | 0.8583 | 0.2265 |
