# Supplementary material for: Intimate partner violence against women in Nigeria: a multilevel study investigating the effect of women’s status and community norms
Source: BMC Womens Health. 2018 Aug 9;18:136. doi: 10.1186/s12905-018-0628-7 (PMC6085661; doi:10.1186/s12905-018-0628-7)
Supplement: Supplementary file 3 — Table S2. Baseline characteristics of the men interviewed. (DOCX 20 kb) [file 12905_2018_628_MOESM3_ESM.docx]

Table S2: Baseline characteristics of the men interviewed.

|  | Does not justify IPV  N (%) | Justifies IPV  N (%) | Total  N (%) |
| --- | --- | --- | --- |
| Age  15 – 24  25 – 34  35 – 44  >44 | 4554 (35.6)  3812 (30.0)  3007 (23.7)  1376 (10.7) | 1857 (41.6)  1302 (29.9)  932 (20.7)  354 (7.8) | 6411 (37.1)  5114 (30.0)  3939 (22.9)  1730 (10.0) |
| Partnership  Single  Monogamous  Polygamous | 6247 (48.2)  5448 (43.3)  1054 (8.5) | 2405 (52.8)  1651 (38.6)  389 (8.6) | 8652 (49.4)  7099 (42.1)  1443 (8.5) |
| Education level  No education  Primary  Secondary  Higher | 2316 (20.2)  2093 (15.9)  6086 (47.4)  2254 (16.5) | 991 (24.1)  862 (19.4)  2218 (48.6)  374 (8.0) | 3307 (21.1)  2955 (16.8)  8304 (47.7)  2628 (14.4) |
| Employment status  Not employed  Employed | 2975 (23.1)  9707 (76.9) | 1062 (23.0)  3366 (77.0) | 4037 (23.1)  13073 (76.9) |
| Place of residence  Urban  Rural | 5646 (47.2)  7103 (52.8) | 1459 (34.6)  2986 (65.4) | 7105 (44.0)  10089 (56.0) |
| Household wealth quintile  Poorest  Poorer  Middle  Richer  Richest | 1733 (14.9)  2064 (15.6)  2525 (18.7)  3039 (22.6)  3388 (28.2) | 876 (20.8)  931 (21.9)  970 (20.8)  980 (21.0)  688 (15.5) | 2609 (16.4)  2995 (17.2)  3495 (19.2)  4019 (22.2)  4076 (25.0) |
| Region  North Central  North East  North West  South East  South South  South West | 2183 (14.3)  1866 (12.5)  3106 (31.3)  1200 (9.7)  2096 (13.6)  2298 (18.7) | 822 (19.3)  955 (20.5)  967 (25.2)  475 (10.1)  883 (15.1)  343 (9.9) | 3005 (15.5)  2821 (14.5)  4073 (29.8)  1675 (9.8)  2979 (14.0)  2641 (16.5) |
| TOTAL | 12749 (75.1) | 4445 (24.9) | 17194 (100) |
