# Supplementary material for: Intimate partner violence against women in Nigeria: a multilevel study investigating the effect of women’s status and community norms
Source: BMC Womens Health. 2018 Aug 9;18:136. doi: 10.1186/s12905-018-0628-7 (PMC6085661; doi:10.1186/s12905-018-0628-7)
Supplement: Supplementary file 5 — Table S3. Single level logistic regression of the association between IPV, women’s status and covariates among ever-partnered women in Nigeria (DOCX 18 kb) [file 12905_2018_628_MOESM5_ESM.docx]

Table S3: Single level logistic regression of the association between IPV, women status and covariates among ever-partnered women in Nigeria

| **Variables** | **Simple regression**  **OR (95% CI)** | **Multivariate regression**  **OR (95% CI)** |
| --- | --- | --- |
| **Women’s status**  Low  Middle  High | 1  1.09 (1.01 – 1.18)  0.95 (0.88 – 1.03) | 1  1.07 (0.96 – 1.18)  0.98 (0.87 – 1.10) |
| **Age group**  15 – 24  25 – 34  35 – 44  45 - 49 | 1  1.37 (1.26 – 1.48)  1.41 (1.29 – 1.55)  1.08 (0.95 – 1.22) | 1  1.26 (1.14 – 1.39)  1.35 (1.21 – 1.50)  1.22 (1.05 – 1.42) |
| **Wealth quintile**  Poorest  Poor  Middle  Richer  Richest | 1  1.53 (1.38 – 1.70)  1.87 (1.68 – 2.07)  1.77 (1.60 – 1.96)  1.51 (1.36 – 1.67) | 1  1.26 (1.11 – 1.43)  1.26 (1.10 – 1.45)  1.07 (0.92 – 1.25)  1.01 (0.85 – 1.21) |
| **Place of residence**  Urban  Rural | 1  0.89 (0.84 – 0.95) | 1  1.03 (0.93 – 1.13) |
| **Nature of Union**  Monogamous  Polygamous | 1  1.05 (0.97 – 1.13) | 1  1.14 (1.04 – 1.25) |
| **Woman’s attitude to IPV**  Does not justify wife-beating  Justifies wife-beating | 1  1.63 (1.53 – 1.74) | 1  1.53 (1.42 – 1.65) |
| **Witnessed mother being beaten**  No  Yes | 1  4.15 (3.77 – 4.57) | 1  2.85 (2.55 – 3.18) |
| **Partner’s alcohol use**  Does not drink  Never gets drunk  Gets drunk sometimes  Gets drunk often | 1  2.32 (2.03 – 2.66)  3.48 (3.19 – 3.79)  8.71 (7.32 – 10.4) | 1  2.05 (1.88 – 2.62)  2.46 (2.19 – 2.71)  6.73 (5.48 – 8.25) |
| **Partner’s controlling behavior**  None  Yes | 1  3.80 (3.51 – 4.12) | 1  3.90 (3.55 – 4.27) |
| **Partner’s education level**  Tertiary  Secondary  Primary  No education | 1  1.41 (1.28 – 1.56)  1.57 (1.41 – 1.75)  0.65 (0.58 – 0.72) | 1  1.16 (1.03 – 1.31)  1.23 (1.06 – 1.42)  0.90 (0.70 – 1.16) |
| **Education difference between partners**  Both partners educated equally  Partner more educated than woman  Woman more educated than partner  Both partners are not educated | 1  1.14 (1.04 – 1.24)  1.25 (1.13 – 1.38)  0.49 (0.45 – 0.54) | 1  1.09 (0.98 – 1.21)  1.15 (1.01 – 1.32)  0.73 (0.57 – 0.92) |
| **Income difference between partners**  Both partners earn equally  Partner earns more than woman  Woman earns more than partner  Both partners do not earn | 1  1.04 (0.88 – 1.22)  1.47 (1.17 – 1.85)  1.01 (0.85 – 1.19) | 1  1.17 (0.96 – 1.42)  1.65 (1.25 – 2.17)  1.24 (1.00 – 1.54) |
