# Supplementary material for: Intimate partner violence against women in Nigeria: a multilevel study investigating the effect of women’s status and community norms
Source: BMC Womens Health. 2018 Aug 9;18:136. doi: 10.1186/s12905-018-0628-7 (PMC6085661; doi:10.1186/s12905-018-0628-7)
Supplement: Supplementary file 6 — Figure S3. Contribution of individual- and community-level to the intra-class correlation (ICC). (DOCX 27 kb) [file 12905_2018_628_MOESM6_ESM.docx]

Figure S3: Contribution of individual- and community-level to the intra-class correlation (ICC).
